# EQ-5D-5L norms for the urban Chinese population in China

**DOI:** 10.1186/s12955-018-1036-2

**Published:** 2018-11-08

**Authors:** Zhihao Yang, Jan Busschbach, Gordon Liu, Nan Luo

**Affiliations:** 1 Health Services Management Department, Guizhou Medical University, No.9 Beijing Road, Guiyang, China; 2 Medical Psychology and Psychotherapy, Erasmus Medical Center, Wytemaweg, 80 Rotterdam, The Netherlands; 3 National School of Development, Peking University, 5 Yiheyuan Road, Beijing, 100871 China; 4 Saw Swee Hock School of Public Health, National University of Singapore, 6 Medical Drive, Block MD3, Singapore, 117597 Singapore

**Keywords:** EQ-5D-5L, Population norm, China, HRQoL

## Abstract

**Background:**

To generate Chinese population norms for the EQ-5D-5L dimensions, EQ-VAS (Visual Analogue Scale) scores and EQ-5D-5L index scores, stratified by gender and age. The EQ-5D is a widely used generic health-related quality of life instrument to describe population health and health outcomes in clinical trials and health economic evaluations. Currently, there are no EQ-5D-5L population norms for China.

**Methods:**

This norm study utilized the data collected in an EQ-5D-5L valuation study in China between December 2012 and January 2013. In the valuation study, respondents were asked to report their own health states using the EQ-5D-5L descriptive system and the EQ-VAS. Respondents’ demographic information was also collected. The EQ index score was calculated using the EQ-5D-5L value set based on the Chinese urban population. Norm scores were reported by important demographic variables.

**Results:**

The mean EQ-VAS scores ranged between 88.3 for males of < 19 years and 82.9 for females of 60–69 years. Contrary to other population studies, females reported higher EQ-VAS scores than males in every age group except for 20–29 years. The mean EQ-5D-5L index values ranged from 0.912 for females of > 70 years to 0.971 for females of 30–39 years. Respondents reported more problems in the dimensions ‘pain/discomfort’ and ‘anxiety/depression’ than in the dimensions ‘mobility’, ‘self-care’ and ‘usual activities’ in all age groups.

**Conclusions:**

The population norm scores for the EQ-5D can be used as reference values for comparative purposes in future Chinese studies. Further research into rural and/or a more representative population is warranted.

## Introduction

EQ-5D is a health-related quality of life (HRQoL) questionnaire widely used in economic, clinical, and population health studies. The EQ-5D descriptive system comprises five dimensions: mobility, self-care, usual activities, pain/discomfort and anxiety/depression [1]. It has two versions, a three-level EQ-5D (EQ-5D-3L) and a five-level EQ-5D (EQ-5D-5L). Although EQ-5D-3L has been widely used, it is reported to suffer from ceiling effects (i.e. the percentage of respondent reporting the best possible health state on EQ-5D) and measurement insensitivity [2]. By increasing the number of levels in the descriptive system, EQ-5D-5L has demonstrated reduced ceiling effects and improved discriminatory power in comparison to EQ-5D-3L [3–7]. In addition to classifying health states in terms of the 5 dimensions of health, EQ-5D permits the valuation of these health states. This is accomplished from both the respondent’s own perspective by using a Visual Analogue Scale (EQ-VAS) and from the perspective of the general public’s by attaching the appropriate EQ-5D index score to the described health state of the respondent.

EQ-5D has been used to measure population health in many countries, and population norms have been established by age, gender and socio-economic status [1]. A set of population norm scores provides an important reference point for clinical and health economic research outcomes, as the effects of medical conditions and/or treatments can be quantified by comparing patients and/or intervention groups with the general population [8]. At this juncture, there are no EQ-5D-5L norms for the Chinese population, which hampers the increasing use of EQ-5D-5L in China.

The objective of this paper is to provide population norm, including the prevalence of EQ-5D-5L health problems, and EQ-VAS and EQ index scores by age and gender, in the Chinese urban population. In addition, we also examine the relationships between socio-economic factors and (i) the components of the EQ-5D-5L descriptive system, (ii) EQ-VAS scores and (iii) EQ index scores.

## Methods

### Sampling and recruitment

This norm study drew data from a large EQ-5D-5L valuation study in China [3]. In the valuation study, respondents reported: 1) valuation data and; 2) self-reported health status data and demographic data. The valuation data was used to establish the EQ-5D-5L value set for China and was reported elsewhere [3]. This study used the self-reported health status data and demographic data to establish a population norm. The sample size was decided by the EQ-5D-5L valuation protocol, which was aiming at constructing country specific EQ-5D-5L value set [9]. Members of the general population were randomly recruited from five urban areas of five cities (Beijing, Shenyang, Nanjing, Chengdu, and Guiyang). From each city, respondents were recruited from at least five difference administrative districts and at different time of day. Specific recruitment sites included library, hospital, university, local community, park and shopping areas etc. [10]. These five cities were selected as representative urban areas in terms of size of population, geographical region and economic development status in China [3]. Within each city, quotas were set to recruit equal numbers of participants from each city and to ensure the study sample resembled the general Chinese urban adult population with respect to age, gender, and education level according to the Sixth National Population Census [10]. In each city, members of the general public who were at least 16 years old, and were literate and able to understand survey questions, were recruited through personal invitation [3]. Response rate was calculated.

Each respondent was interviewed face-to-face by a trained interviewer using the EuroQol valuation technology (EQ-VT) [3, 11]. EQ-VT is a standardized software design by the EuroQol Group in order to facilitate the data collection for valuation study [9]. The interview had four sections. The first section was for respondents to report their own health using the EQ-5D-5L questionnaire: the five-dimensional descriptive system and the EQ-VAS. In the second section respondents were asked to value 10 different EQ-5D-5L health states using a composite time trade-off (cTTO) method [8]. The third section contained 7 pairs of EQ-5D-5L discrete choice tasks. The fourth section assessed respondents’ socio-economic and other background characteristics. This paper used data collected in the first and fourth sections only.

### The EQ-5D questionnaire

The EQ-5D-5L descriptive system consists of five dimensions (mobility, self-care, usual activities, pain/ discomfort and anxiety/depression) with five ordinal severity levels each (no problems, slight problems, moderate problems, severe problems, and extreme problems/unable to), thus defining 3125 (5^5^) distinct health states [2]. The respondent is asked to indicate his/her health state against the most appropriate statement in each of the 5 dimensions and this leads to a 1-digit number expressing the level selected for each dimension [1], i.e. 12211 means the respondent had no problems in mobility, pain/discomfort, and anxiety/depression, but had slight problems in self-care and usual activities. A VAS was used in the interview, with anchor points 0 (‘worst imaginable health state’) and 100 (‘best imaginable health state’). Respondents first report their own health state using the EQ-5D-5L descriptive system and then their overall health on the EQ-VAS based on their health on the day of survey.

In 2012, the Chinese version of EQ-5D-5L was translated using a response scaling method by Luo et al. [2].The translation process followed the standard translation protocol developed by the EuroQol Group and had mainly 3 stages [2]. First, different candidate labels were generated by direct translation and reviewing existing questionnaire; second, 50 native Chinese speaking respondents were interviewed to rank and value potential candidates labels; third, the final labels were selected to achieve comparable measurement properties to the English/Spanish version of EQ-5D-5L. The translated Chinese EQ-5D-5L demonstrated validity and increased sensitivity in diabetes and hepatitis B patients [12, 13].

### Data analysis

For each respondent, the EQ-5D-5L health state and the EQ-VAS were directly observed from respondent’s’ self-report questionnaire while the EQ index score was derived from the Chinese EQ-5D-5L value set [3]. In the EQ-5D-5L value set, the EQ index score of all 3125 health states were estimated [3]. For each respondent, we derived their corresponding EQ index score from their self-reported health states.

First, descriptive statistics of EQ-5D-5L health state, EQ-VAS and EQ index score were calculated for the whole sample and by different demographic variables and cities (age, gender, employment status etc.). For comparison, we categorized age into age groups following other countries’ population norm studies [6, 14–17]. As our sample was recruited from urban areas only, we used ‘Residence of origin’ as a proxy to study the possible difference of HRQoL between urban and rural residents. ‘Residence of origin’ referred to the birth place of the respondent and was classified into three groups (city, county, township or village) based on China’s administrative levels. ‘Health insurance’ referred to social health insurance types in China’s healthcare system [18]. For each demographic variable, the percentage of reported problem in EQ-5D dimension, the means (and 95% confidence interval) of EQ-VAS and EQ index scores were calculated for each subgroup and the difference were tested statistically. Second, we used multivariable analysis to examine the associations between demographic characteristics with reported problems in EQ-5D-5L, EQ-VAS and EQ index scores respectively. For the reported problems in each dimension, we used logistic regression (‘no problems’ coded as 0; ‘slight problems’, ‘moderate problems’, ‘severe problems’, or ‘extreme problems/unable’ coded as 1) [1]. For EQ-VAS and EQ index scores, we used linear regression. All demographic variables including age and education level were entered into the models as categorical variables. For better interpretation, age groups were collapsed into three categories (young age: <=29 years, middle age: 30–59 years; older age: > = 60 years) [14]. Multivariable analysis was used to identify significant demographic characteristics using a backward selection procedure to remove covariates with *p* > 0.05. Odds ratio was reported for logistic regression and coefficient was reported for linear regression respectively, the corresponding 95% CI was calculated using robust standard error.

For this study, ethical approval was not needed in China at the time of data collection. A waiver of the informed consent was approved as this study did not provide any intervention to participants. Participants can withdraw at any time without any consequences.

## Results

A total of 1332 individuals (response rate: 68.6%) who met the inclusion criteria were recruited. Among these, 1296 (97.3%) who successfully completed the questionnaire were included in the analysis. The mean age of the sample was 42 years (SD: 16 years), the age ranged between 16 years to 85 years old. Females comprised 49.9% of the sample. Other demographic information is shown in Table 1.

**Table 1:** Demographic characteristics of all respondents

	Our sample	
Age group, years	N	%
16–19	109	8.4
20–29	229	17.7
30–39	244	18.8
40–49	272	21.0
50–59	220	17.0
60–69	155	12.0
> 70	67	5.2
Gender		
Female	646	49.9
Male	650	50.2
Education		
Primary or lower	138	10.7
Junior & Senior high school	867	66.9
College or higher	291	22.5
Employment status		
Full time employees	382	29.5
Temporary worker & freelancer	451	34.8
Retired	240	18.5
Student	132	10.2
Other	91	7.0
Residence of origin		
City	757	58.4
County	86	6.6
Township or village	453	35.0
Health insurance		
Urban employee	551	42.5
Urban residence	304	23.5
New rural	296	22.8
Other	88	6.8
No	57	4.4

In total, 54% of the sample reported their health as ‘11111’, followed by ‘11121’, ‘11112’, ‘11122’, and ‘21121’. The percentages of ‘no problems’ were: 94.37% for mobility, 98.92% for self-care, 95.45% for usual activity, 70.14% for pain/discomfort, and 73.15% for anxiety/depression. The mean EQ-VAS and EQ index scores were 86.0 (SD: 11.4) and 0.957 (SD: 0.069), respectively.

Tables 2 and 3 show the percentage of reported problems for each severity level and EQ-5D dimension, and the mean (SD) of EQ-VAS and EQ index scores for males and females by age groups, respectively. In both male and female groups, the number of problems increased with age in the dimensions of mobility, self-care, and pain/discomfort (*p* < 0.05, trend test for ordered groups). In contrast, anxiety/depression was more prevalent in younger age groups (*p* < 0.01, trend test for ordered groups). As could be expected, the means of both EQ-VAS and EQ index scores decreased with age, but only the EQ index score for male was statistically significant (*p* < 0.05, trend test for ordered groups). Females reported higher EQ-VAS values than males (p < 0.05, two-sample t-test). The highest mean EQ index score was observed for females of 30–39 years (0.971), the lowest mean score for females of > 70 years (0.912). The mean VAS score ranged between 88.3 for females of < 19 years and 82.9 for males of 60–69 years.

**Table 2:** Percentage of a general population sample reporting levels 1 to 5 by dimension, EQ-VAS & EQ index score by age group for males

EQ-5D dimension		Age Groups							Total *N* = 650
		16–19‚ *N* = 56	20–29‚ *N* = 116	30–39‚ *N* = 123	40–49‚ *N* = 135	50–59‚ *N* = 110	60–69‚ *N* = 84	> 70‚ *N* = 26	
Mobility	No problems	100%	98.3%	98.4%	91.9%	96.4%	85.7%	69.2%	94.0%
	Slight problems	0%	1.7%	1.6%	8.2%	3.6%	13.1%	26.9%	5.7%
	Moderate problems	0%	0%	0%	0%	0%	1.2%	3.9%	0.3%
	Severe problems	0%	0%	0%	0%	0%	0%	0%	0%
	Unable to	0%	0%	0%	0%	0%	0%	0%	0%
	Z (*P* value)							5.69 (0.000)	
Self-care	No problems	100%	100%	100%	98.5%	100%	96.4%	96.2%	99.1%
	Slight problems	0%	0%	0%	1.5%	0%	3.6%	3.9%	0.9%
	Moderate problems	0%	0%	0%	0%	0%	0%	0%	0%
	Severe problems	0%	0%	0%	0%	0%	0%	0%	0%
	Unable to	0%	0%	0%	0%	0%	0%	0%	0%
	Z (P value)							2.65 (0.008)	
Usual Activity	No problems	96.4%	94.8%	95.9%	93.3%	99.1%	90.5%	92.3%	94.9%
	Slight problems	3.6%	5.2%	4.1%	5.9%	0.9%	7.1%	7.7%	4.6%
	Moderate problems	0%	0%	0%	0.7%	0%	2.4%	0%	0.5%
	Severe problems	0%	0%	0%	0%	0%	0%	0%	0%
	Unable to	0%	0%	0%	0%	0%	0%	0%	0%
	Z (P value)							0.95 (0.342)	
Pain/Discomfort	No problems	78.6%	75.9%	78.1%	71.1%	64.6%	64.3%	57.7%	71.4%
	Slight problems	19.6%	23.3%	20.3%	26.7%	29.1%	31.0%	30.8%	25.4%
	Moderate problems	1.8%	0%	0.8%	1.5%	6.4%	4.8%	11.5%	2.8%
	Severe problems	0%	0.9%	0.8%	0.7%	0%	0%	0%	0.5%
	Extreme problems	0%	0%	0%	0%	0%	0%	0%	0%
	Z (P value)							3.44 (0.001)	
Anxiety/Depression	No problems	67.9%	65.5%	66.7%	78.5%	75.5%	77.4%	88.5%	72.8%
	Slight problems	30.4%	32.8%	29.3%	20.7%	21.8%	20.2%	11.5%	25.1%
	Moderate problems	1.8%	1.7%	2.4%	0%	1.8%	0%	0%	1.2%
	Severe problems	0%	0%	0.8%	0.7%	0.9%	2.4%	0%	0.6%
	Extreme problems	0%	0%	0.8%	0%	0%	0%	0%	0.3%
	Z (P value)							−2.94 (0.003)	
EQ-VAS	Mean	87.4	86.9	85.5	85.5	84.8	82.9	83.9	85.4
	95%CI	84.4	85.2	83.8	83.3	82.6	79.9	76.9	84.5
		90.4	88.5	87.2	87.8	87.1	85.9	90.9	86.3
	Z (P value)							−1.68 (0.093)	
EQ index score	Mean	0.968	0.963	0.961	0.959	0.956	0.943	0.932	0.957
	95%CI	0.957	0.953	0.950	0.948	0.946	0.921	0.897	0.952
		0.978	0.973	0.972	0.971	0.967	0.964	0.966	0.962
	Z (P value)							−2.21 (0.027)

**Table 3:** Percentage of a general population sample reporting levels 1 to 5 by dimension, EQ-VAS & EQ index score by age group for females

EQ-5D dimension		Age Groups							Total *N* = 646
		16–19‚ *N* = 53	20–29‚ *N* = 113	30–39‚ *N* = 121	40–49‚ *N* = 137	50–59‚ N = 110	60–69‚ *N* = 71	> = 70‚ *N* = 41	
Mobility	No problems	96.2%	96.5%	99.2%	97.1%	95.5%	90.1%	73.2%	94.7%
	Slight problems	3.8%	3.5%	0.8%	2.9%	3.6%	8.5%	19.5%	4.5%
	Moderate problems	0%	0%	0%	0%	0%	1.4%	7.3%	0.6%
	Severe problems	0%	0%	0%	0%	0.9%	0%	0%	0.2%
	Unable to	0%	0%	0%	0%	0%	0%	0%	0%
	Z (P value)							4.68 (0.000)	
Self-care	No problems	98.1%	99.1%	99.2%	100%	99.1%	97.2%	95.1%	98.8%
	Slight problems	1.9%	0.9%	0.8%	0%	0%	1.4%	4.9%	0.9%
	Moderate problems	0%	0%	0%	0%	0%	1.4%	0%	0.2%
	Severe problems	0%	0%	0%	0%	0.9%	0%	0%	0.2%
	Unable to	0%	0%	0%	0%	0%	0%	0%	0%
	Z (P value)							1.42 (0.156)	
Usual Activity	No problems	96.2%	99.1%	98.4%	97.8%	96.4%	93.0%	78.1%	96.0%
	Slight problems	3.8%	0.9%	1.7%	2.2%	1.8%	7.0%	22.0%	3.7%
	Moderate problems	0%	0%	0%	0%	0.9%	0%	0%	0.2%
	Severe problems	0%	0%	0%	0%	0.9%	0%	0%	0.2%
	Unable to	0%	0%	0%	0%	0%	0%	0%	0%
	Z (P value)							4.36 (0.000)	
Pain/Discomfort	No problems	66.0%	74.3%	76.0%	69.3%	65.5%	64.8%	51.2%	68.9%
	Slight problems	30.2%	24.8%	23.1%	28.5%	32.7%	32.4%	39.0%	28.8%
	Moderate problems	1.9%	0.9%	0.8%	1.5%	0.9%	2.8%	7.3%	1.7%
	Severe problems	1.9%	0%	0%	0.7%	0.9%	0%	2.4%	0.5%
	Extreme problems	0%	0%	0%	0%	0%	0%	0%	0.2%
	Z (P value)							2.56 (0.010)	
Anxiety/Depression	No problems	56.6%	62.8%	76.9%	75.9%	76.4%	85.9%	78.1%	73.5%
	Slight problems	37.7%	31.9%	20.7%	21.9%	21.8%	14.1%	19.5%	23.7%
	Moderate problems	5.7%	4.4%	2.5%	1.5%	1.8%	0%	2.4%	2.5%
	Severe problems	0%	0.9%	0%	0%	0%	0%	0%	0.2%
	Extreme problems	0%	0%	0%	0.7%	0%	0%	0%	0.2%
	Z (P value)							−4.02 (0.000)	
EQ-VAS	Mean	88.3	85.8	87.8	87.5	86.2	84.5	85.3	86.6
	95%CI	85.4 91.2	83.6 88.0	86.0 89.6	85.6 89.3	84.0 88.3	81.8 87.2	82.0 88.6	85.8 87.5
	Z (P value)							−1.75 (0.081)	
EQ index score	Mean	0.945	0.959	0.971	0.962	0.954	0.957	0.912	0.957
	95%CI	0.926 0.963	0.949 0.968	0.962 0.979	0.952 0.972	0.933 0.975	0.943 0.971	0.881 0.943	0.951 0.962
	Z (P value)							−1.04 (0.300)	

Beside age and gender, Table 4 shows the percentage of any reported problem for each EQ-5D dimension, and the mean (SD) of EQ-VAS and EQ index scores by other socio-demographic characteristics. Lower education indicated more problems in mobility, usual activities and more pain (*p* < 0.05, chi^2^ test). Lower education also had lower EQ index score (p < 0.05, one-way analysis of variance). Percentage of any reported problem all differed by employment status (*p* < 0.01, chi^2^ test), full time employees reported least problems with self-care and usual activities; students reported the least problems with mobility and less pain/discomfort; retired reported least anxiety/depression. Students reported the highest score in EQ-VAS and EQ index score. Percentage of reported problem in any dimensions did not differ between the insured respondents and respondents without insurance, but the EQ-VAS of the insured was higher than those without insurance (*p* < 0.05, two-sample t-test). In terms of original place of residence, residents from the city reported less anxiety (*p* < 0.01, chi^2^ test). Difference were also found between cities in pain/discomfort, anxiety/depression, EQ-VAS and EQ index score.

**Table 4:** Percentage of a general population sample reporting any problem by dimension, EQ-VAS & EQ index score by other demographic variables

	Mobility	Self-care	Usual activities	Pain/discomfort	Anxiety/depression	EQ-VAS (95%CI)	EQ-index (95%CI)
Highest education							
Primary school & lower(*n* = 138)	10.9%	1.4%	9.4%	37.0%	25.4%	84.8 (82.9, 86.8)	0.943 (0.924, 0.961)
High school(*n* = 867)	5.4%	0.9%	4.1%	30.3%	24.6%	86.2 (85.5, 87.0)	0.959 (0.954, 0.963)
College & above(*n* = 291)	3.8%	1.0%	3.4%	25.1%	34.4%	85.9 (84.7, 87.0)	0.959 (0.952, 0.965)
P value	0.01	0.91	0.01	0.04	0.00	0.40	0.04
Employment status							
Full time employee(*n* = 382)	2.6%	0%	1.8%	28.3%	29.1%	87.5 (86.6, 88.5)	0.963 (0.957, 0.968)
Part time & freelancer(*n* = 451)	4.2%	0.7%	4.7%	29.3%	26.6%	85.6 (84.5, 86.7)	0.960 (0.955, 0.966)
Retired(*n* = 240)	13.3%	2.9%	7.1%	37.5%	16.7%	83.8 (82.2, 85.5)	0.948 (0.937, 0.958)
Student(*n* = 132)	1.5%	0.8%	3.0%	17.4%	40.1%	88.7 (87.3, 90.0)	0.964 (0.957, 0.972)
Others(*n* = 91)	11.0%	3.3%	11.0%	37.4%	26.4%	83.7 (80.7, 86.8)	0.930 (0.902, 0.957)
P value	0.00	0.00	0.00	0.00	0.00	0.00	0.00
Health Insurance							
With insurance (*n* = 1239)	5.7%	1.1%	4.4%	29.9%	26.8%	86.1 (85.5, 86.8)	0.957 (0.953, 0.961)
Without insurance (*n* = 57)	3.5%	0%	7.0%	29.8%	28.1%	82.8 (79.4, 86.1)	0.953 (0.933, 0.974)
P value	0.48	0.42	0.36	1.00	0.83	0.03	0.71
Residence of origin							
City(*n* = 757)	6.7%	1.1%	4.1%	31.2%	23.7%	85.6 (84.8, 86.5)	0.957 (0.952, 0.962)
County(n = 86)	5.8%	1.2%	8.1%	32.6%	34.9%	85.4 (83.1, 87.7)	0.952 (0.941, 0.964)
Township or village(*n* = 453)	3.8%	1.1%	4.6%	27.2%	30.7%	86.8 (85.8, 87.8)	0.957 (0.950, 0.965)
P value	0.09	0.99	0.23	0.29	0.00	0.19	0.82
Cities							
Beijing	3.0%	0%	2.3%	28.2%	17.9%	88.5 (87.4, 89.7)	0.968 (0.962, 0.974)
Chengdu	6.6%	1.2%	6.3%	34.8%	31.6%	84.9 (83.4, 86.5)	0.949 (0.941, 0.957)
Guiyang	7.7%	1.2%	5.8%	21.8%	28.0%	86.0 (84.7, 87.2)	0.959 (0.949, 0.969)
Nanjing	5.6%	1.5%	4.5%	36.7%	34.1%	85.2 (83.8, 86.6)	0.948 (0.939, 0.956)
Shenyang	5.2%	1.6%	4.0%	27.6%	22.4%	85.4 (83.8, 87.0)	0.961 (0.952, 0.969)
P value	0.21	0.41	0.21	0.00	0.00	0.00	0.00

Socio-demographic characteristics which significantly predicted any problems in EQ-5D dimensions, and EQ-VAS and EQ index scores, are reported in Table 5, where the reported problem in each dimension was reported as an odds ratio, and the EQ-VAS, EQ index scores were reported as regression coefficients. Notably, reported problems with anxiety/depression declined along age groups (odds ratio: 0.58 for 30–59 years; 0.40 for > = 60 years respectively). Males had 1.45 lower EQ-VAS value than females. All outcomes varied with employment status. For example, compared to the group with full time job, unemployed group reported 4.04 lower EQ-VAS value and 0.03 lower EQ-index score, retired group reported 3.93 lower EQ-VAS value and 0.02 lower EQ-index score. Respondents from the county were found more reported problem in usual activities (odds ratio: 2.58).

**Table 5:** The association between HRQoL data and demographic factors (*N* = 1296)

Variables	Mobility	Self-care	Usual activity	Pain /discomfort	Anxiety /depression	EQ-VAS	EQ-index score
	Odds Ratio 95%CI					Coefficients 95%CI	
Age group (Ref: <=29 years group)	1.00	1.00	1.00	1.00	1.00		
30–59 years groups	1.14		0.87		**0.58 (0.44,0.77)**		
> =60 years groups	**4.89 (1.94,12.32)**		**3.67 (1.40,9.60)**		**0.40 (0.26,0.59)**		
Gender (Ref: female)	1.00	1.00	1.00	1.00	1.00		
Male						**−1.45 (−2.69,-0.22)**	
Health Insurance (Ref: no insurance)	1.00	1.00	1.00	1.00	1.00		
With Insurance						**3.36 (0.08,6.63)**	
Employment status (Ref: full time job)	1.00	1.00	1.00	1.00	1.00		
Temporary worker& freelancer	1.48	**0.20** **(0.04,0.99)**	2.31	1.05		**−1.84 (−3.30,-0.39)**	0.00
Retired	1.83	0.88	1.55	**1.52 (1.08,2.15)**		**−3.93 (−7.22,-0.85)**	**− 0.02 (− 0.03,-0.00)**
Student	0.65	0.22	1.52	**0.54 (0.32,0.88)**		0.98	0.00
Unemployed & others	**3.05 (1.22,7.62)**	1.00	**4.54 (1.74,11.87)**	1.51		**−4.04 (−6.63,-1.45)**	**−0.03 (−0.06,-0.00)**
Residence of origin (Ref: city)	1.00	1.00	1.00	1.00	1.00		
County			**2.58 (1.03,6.48)**				
Village			1.19				

Note: *CI*: Confidence interval, Bolded: statistically significant; 95%CIs were calculated using robust standard error

## Discussion

This is the first EQ-5D-5L norms study from China. These general population-based norms provide insights into HRQoL in China and how HRQoL varies between different socio-economic groups. More importantly, it facilitates interpretation of the cost effectiveness studies which use QALY as a health outcome. As HRQoL instruments measure postulated constructs, the set of norm scores provides a reference point to interpret an HRQoL study’s results by comparing HRQoL between the general population and patients with specific conditions from similar age and gender groups [19, 20].

Compared to the Chinese EQ-5D-3L norms reported in 2008 [21], our study showed a significant increase in problems reported in the last two dimensions. This could be either because there were more problems in these two dimensions compared to the past, or that the five-level EQ-5D was more sensitive in identifying the mild problems in these dimensions. While it is not possible to detangle such change in our study, in several studies comparing norm scores between EQ-5D-3L and EQ-5D-5L, the researchers reported the 5L questionnaire suffered less ceiling effect, had less standard deviation in the index value, and had wider spread of health states, which all suggests the improved sensitivity for the 5L questionnaire [4, 13, 16]. HRQoL inequalities were shown in China between different socio-demographic groups and regions, based on previous research [21–24]. Such disparities were confirmed by our multivariable analysis, with lower socio-economic status related to lower HRQoL.

Some results from our study were in line with other countries’ EQ-5D-5L norms [4, 6, 13–17, 25]: the first three dimensions of EQ-5D had less reported problems compared to the last two dimensions, with pain/discomfort being the most prevalent dimension; women reported lower EQ index score than men; EQ-VAS and EQ index score declined with age. Two differences were noted, first, in previous EQ-5D norms studies conducted in China and other countries, the percentage of reported problems in anxiety/depression increased with age [1, 4, 6, 13, 25, 26], our results suggest the opposite: the anxiety/depression problem was more prevalent in the younger population. One possible explanation is that the younger generation living in urbans areas perceived more psychological pressures than the older generation due to the fast-paced life in urban China. Second, females reported slightly higher EQ-VAS values than males, which is inconsistent with EQ-5D-3L norm values in China [21]: this discrepancy could be due to the difference in the two study samples’ compositions. The EQ-VAS score is predicted by several demographic variables and in our study sample, females were in higher socio-economic groups.

One limitation of this study is that the sample was collected in five urban areas in China, which is not representative of the whole Chinese population. As socio-economic differences exist between different areas, also between urban and rural areas in China, the health status of residents may differ by type of area [27]. Furthermore, most respondents were recruited in public locations, therefore the sample may have left out those who were not able to go outside. This may have led to a selection bias towards healthy respondents and underreported problems with mobility and usual activities. Nevertheless, we did not correct for this bias in our result as we did not know the exact proportion of respondents missed out in the sample. Third, this is a cross-sectional study, which provided insights into relationship between HRQoL data and socio-demographic variables. In terms of understanding the causal relationship between variables and controlling for unobserved heterogeneity, longitudinal data is needed [28–30].

## Conclusions

This study has offered the first EQ-5D-5L urban population norms for China. Disparities exist in self-reported health status measured by EQ-5D-5L across socio-economic groups. Further research into rural HRQoL and into using a national representative sample is warranted.
